# Evaluation of Nutritional Status and the Impact of Nutritional Treatment in Patients with Pancreatic Cancer

**DOI:** 10.3390/cancers15153816

**Published:** 2023-07-27

**Authors:** Dominika Mękal, Jacek Sobocki, Anna Badowska-Kozakiewicz, Katarzyna Sygit, Elżbieta Cipora, Ewa Bandurska, Aleksandra Czerw, Andrzej Deptała

**Affiliations:** 1 Department of Oncology Propaedeutics, Medical University of Warsaw, 01-445 Warsaw, Poland; anna.badowska-kozakiewicz@wum.edu.pl (A.B.-K.); andrzej.deptala@wum.edu.pl (A.D.); 2 Department of General Surgery and Clinical Nutrition, Centre for Postgraduate Medical Education, 01-813 Warsaw, Poland; sobockij@gmail.com; 3 Faculty of Health Sciences, Calisia University, 62-800 Kalisz, Poland; ksygit@poczta.onet.pl; 4 Medical Institute, Jan Grodek State University, 38-500 Sanok, Poland; ecipora@up-sanok.edu.pl; 5 Center for Competence Development, Integrated Care and e-Health, Medical University of Gdansk, 80-204 Gdansk, Poland; ewa.bandurska@gumed.edu.pl; 6 Department of Health Economics and Medical Law, Medical University of Warsaw, 01-445 Warsaw, Poland; aleksandra.czerw@wum.edu.pl; 7 Department of Economic and System Analyses, National Institute of Public Health NIH-National Research Institute, 00-791 Warsaw, Poland

**Keywords:** pancreatic cancer, nutritional treatment, malnutrition, cancer cachexia, oncology nutrition

## Abstract

**Simple Summary:**

Malnutrition, cachexia, and sarcopenia are very common problems in PC patients and are associated with an increased risk of chemotherapy-related toxicity, shorter survival, and reduced quality of life (QoL). Approximately 80% of PC patients report weight loss at diagnosis, and 70.3% of patients develop malnutrition during chemotherapy (CT). Early diagnosis of nutritional problems is the first key point in the proper management of nutritional treatment in patients with pancreatic cancer. Healthcare managers, healthcare professionals, PC patients, and their families should be aware of the importance of nutritional status and the role of nutritional management in clinical outcomes and the quality of life of PC patients.

**Abstract:**

Patients with pancreatic cancer who develop irreversible cancer cachexia have a life expectancy of less than 3 months. Therefore, it is extremely important to evaluate the patient’s nutritional status as early as possible and to implement an appropriate nutritional intervention in order to reduce the risk of further weight loss and/or muscle loss, which affect the outcomes of cancer treatment and the correct nutritional treatment in patients with pancreatic cancer. A literature review was performed by using the PubMed and Cochrane quick search methodology. The main purpose of this review was to present the current approach to nutritional treatment in pancreatic cancer. The review included publications, most of which concerned clinical nutrition as part of the phase of treatment of patients with pancreatic cancer, nutritional and metabolic disorders in pancreatic cancer, and the period after pancreatic resection. Some of the publications concerned various nutritional interventions in patients with pancreatic cancer undergoing chemotherapy or surgical treatment (nutritional support before surgery, after surgery, or during palliative treatment). There is an unmet need for integrated nutritional therapy as a key part of the comprehensive care process for PC patients. Nutritional counseling is the first line of nutritional treatment for malnourished cancer patients, but pancreatic enzyme replacement therapy also constitutes the cornerstone of nutritional treatment for relieving symptoms of indigestion and maintaining or improving nutritional status.

## 1. Introduction

In 2020, with 495,773 new cases, pancreatic cancer (PC) was the 12th most common cancer in the world and the seventh leading cause of cancer-related deaths [1]. The prevalence is higher in industrialized countries than in developing countries, suggesting that environmental factors play a key role as risk factors for the disease [2]. Lack of physical activity, obesity, chronic pancreatitis, diabetes, smoking, alcohol consumption, and metabolic syndrome are considered risk factors for the development of PC [2,3].

Malnutrition is a common problem among patients with pancreatic cancer (PC), and this negatively affects their quality of life (QoL) and the effectiveness of treatment. The main purpose of this article is to present the current state of knowledge on nutritional treatment in pancreatic cancer and to analyze various nutritional interventions affecting clinical outcomes in PC patients.

No recommendations and guidelines for nutritional treatment for patients with pancreatic cancer have been developed so far, despite the fact that nutritional intervention in PC patients should be a routine procedure. Nevertheless, there is an unmet need to integrate nutritional treatment as a key part of the multidisciplinary care process for PC patients. Healthcare professionals, cancer patients, and their families should be aware of the importance of nutritional status and medical nutrition therapy (MNT) for clinical outcomes and the quality of life of PC patients.

Under physiological conditions, the pancreas performs both endocrine and exocrine functions. The endocrine function of the pancreas regulates metabolism in the body through the production of insulin, glucagon, and other hormones, while the exocrine function is primarily responsible for the production of enzymes that are necessary for the digestion of fats, carbohydrates, and proteins [4]. Pancreatic exocrine insufficiency (PEI) manifests as nutrient malabsorption accompanied by jaundice, unintentional weight loss, anorexia, epigastric pain, early satiety, and symptoms such as nausea, involuntary vomiting, dehydration, diarrhea, and steatorrhea, and, consequently, protein–energy malnutrition, while hormonal failure most often leads to the development of diabetes [5,6].

Unintentional weight loss is usually the first symptom of pancreatic cancer. Several studies have shown that it affects 50–80% of cancer patients, is a factor depending on the location, stage, and type of tumor, and is closely related to malnutrition and cancer cachexia [7,8]. Pancreatic cancer is one of cancers with a higher incidence of weight loss compared to other cancers [9].

Malnutrition and sarcopenia are common problems in PC patients and result from the advanced stage of the disease and increased toxicity of chemotherapy, resulting in shorter survival and reduced quality of life (QoL) [10,11,12]. Approximately 80% of PC patients report weight loss at diagnosis. More than a third of patients lose >10% of their body weight. Moreover, 70.3% of patients develop malnutrition during chemotherapy (CT) [10].

Malnutrition in cancer patients is multifactorial and results from, among other things, reduced energy and protein intake and increased energy demand of the body. It is accompanied by a progressive loss of muscle and bone mass and, consequently, a decrease in cognitive functions and an increased incidence of other diseases [9].

Cancer-related malnutrition is defined as a multifactorial syndrome characterized by progressive loss of skeletal muscle mass (sarcopenia) with the loss (less often without loss) of adipose tissue, which cannot be fully reversed by conventional nutritional support [13]. Depending on the severity of malnutrition, the following syndromes have been distinguished and are presented in Table 1.

A prospective multicenter cohort study showed that as many as 71% of patients with pancreatic cancer had cachexia at diagnosis, but only 56% of them received nutritional counseling [14]. Therefore, an important goal among patients with pancreatic cancer is to improve their nutritional status and prevent cachexia and malnutrition [15]. The diagnosis of patients at risk of malnutrition and cancer cachexia and the provision of early and appropriate nutritional support are mandatory in all patients with pancreatic cancer starting from the early stages of the disease. It may be beneficial and may affect the outcome of treatment and the quality of life of patients.

To guarantee the best possible effectiveness, appropriate multidisciplinary cooperation of various specialists (e.g., physicians, clinical nutritionists, psycho-oncologists, and nurses) with specific skills and training in the field of nutritional treatment of cancer patients is required.

## 2. Causes and Pathomechanism of Malnutrition in Patients with PC

Malnutrition in patients with pancreatic cancer is multifactorial and is associated with pain, malabsorption, and complex psychological and social factors.

The mechanisms involved in malnutrition associated with pancreatic cancer are numerous and can be divided into catabolic effects due to inflammation, energy, and other nutrient losses resulting from pancreatic dysfunction and anatomical changes resulting from cancer and the side effects of surgical and medical procedures [16].

External compression caused by the tumor or its surgical resection may cause anatomical changes, including mechanical obstruction of the gastrointestinal tract, thus leading to pain or symptoms that affect nutrient intake or absorption, e.g., fatigue, dysphagia, gastroparesis, constipation, and pancreatic insufficiency, leading to exocrine (steatorrhea) and endocrine (diabetes) disorders; this may disrupt the energy balance by increasing the loss of nutrients (mainly fatty acids). PC causes narrowing of the duodenum or stomach by tumor infiltration or compression, leading to intestinal obstruction (clinically manifested by nausea and vomiting). In patients with tumors located in the head of the pancreas, infiltration or compression of the intrapancreatic common bile duct leads to jaundice and numerous disorders of bile secretion and bile flow to the duodenum. This is due to reduced fat digestion and reduced absorption of fat-soluble vitamins. Advanced jaundice leads to liver failure. Moreover, in both proximal and distal tumor locations, exocrine pancreatic insufficiency, which is secondary to pancreatic duct insufficiency, leads to indigestion and malabsorption of all nutrients. In addition, surgical resection of the tumor on the head of the pancreas, i.e., pancreatic duodenotomy, may exacerbate pancreatic insufficiency and reduce oral food intake [4].

In addition, chemotherapy, which is the core of treatment in PC patients, causes side effects such as nausea, anorexia, and vomiting, thus contributing to weight loss and sarcopenia. In addition, radiotherapy may cause side effects in patients with pancreatic cancer, i.e., diarrhea, constipation, abdominal pain, problems with the absorption of nutrients, and others, which may also worsen the nutritional status of patients and increase the risk of malnutrition in this group of patients. In patients with pancreatic cancer, postoperative changes in nutritional status also occur. Surgery of the pancreas significantly affects the pancreatic function and the nutritional status of patients. Patients undergoing pancreaticoduodenectomy (PD) may develop complications such as pancreatic fistula, delayed gastric emptying, dumping syndrome, weight loss, and nutritional deficiencies [16].

Vitamin deficiencies occurring after PD due to bowel resection, altered anatomy of the gastrointestinal tract, and insufficient levels of pancreatic enzymes may also contribute to the deterioration of the nutritional status of patients. Patients are at high risk of vitamin B12 deficiency and often require monthly injections [4].

An important role in the development of cancer cachexia is played by inflammation and its catabolic consequences. The catabolic effects resulting from inflammation influence weight loss and the development of sarcopenia and mediate several cytokines released by the cancer itself and the patient’s immune system. In PC cachexia, interleukin-1 (IL-1), IL-6, IL-8, and tumor necrosis factor ᾳ (TNF-ᾳ) were found to be the most involved factors. In particular, TNF-ᾳ has been highlighted as a major pro-cachectic factor involved in lipolysis, proteolysis, insulin resistance, and muscular dystrophy [16].

Chronic inflammation exerts its effects by interfering with the functions of several tissues as well. First of all, chronic inflammation contributes to the damage of β-pancreatic cells, which can lead to changes in insulin metabolism. In addition, IL-1 has been shown to regulate hypothalamic serotonin release in experimental models. Serotonin, in turn, contributes to the constitutional activation of POMC/CART (cocaine- and amphetamine-regulated transcript) neurons, resulting in an anorexic effect and decreased appetite [16].

Pancreatic exocrine insufficiency (PEI) may also contribute to the development and severity of malnutrition in pancreatic cancer. It is characterized by a deficiency of pancreatic enzymes that are necessary for the absorption of fat, fat-soluble vitamins, and antioxidants, thus causing digestive disorders. Proper exocrine activity of the pancreas is characterized by the production of pancreatic juices consisting of pancreatic enzymes, i.e., pancreatic amylases and lipases, nucleases, and proteases, i.e., trypsin, chymotrypsin, elastase, and carboxypeptidase.

PEI can develop at various stages of cancer: at the beginning of the disease, when the primary location of the tumor is still unknown, after surgery, or during chemotherapy at advanced stages. The incidence of PEI after pancreatic cancer surgery is reported in 50–100% of patients after pancreaticoduodenectomy (PD) and in 0–42% with distal pancreatectomy (DP) [4].

Finally, it should be noted that patients with PC may also exhibit hormonal failure of the pancreas that results in diabetes, referred to as pancreatic diabetes. About 50% of patients have insulin deficiency or diabetes at the time of cancer diagnosis.

## 3. Methods of the Literature Research

The PUBMED database was searched to identify studies on nutritional assessment tools and nutritional management in patients with pancreatic cancer, as well as nutritional interventions before and after pancreatic resection. The search terms included nutritional assessment tools and pancreatic cancer, assessment of nutritional status and pancreatic cancer, nutritional treatment and pancreatic cancer, and nutrition in pancreatic cancer. The inclusion criteria for the review included manuscripts containing primary data on nutritional assessment tools, assessments of nutritional status, malnutrition, cancer cachexia and nutritional treatment, and nutrition of patients diagnosed with pancreatic cancer, a description of the causes of malnutrition and the exocrine and/or endocrine functions of the pancreas, and data on nutritional interventions/treatments ranging from nutritional counseling/ONS, energy, and other nutrient needs to enteral and parenteral nutrition. We excluded case reports, editorials, and manuscripts that focused on other gastrointestinal cancers. Two authors assessed summaries of the publications, qualifying them for further analysis. The potentially eligible publications were then independently assessed by two other authors.

## 4. Assessment of Nutritional Status

Detecting the early signs of malnutrition is crucial not only at the time of diagnosis, but also at every stage of treatment. Various nutritional screening tools have been validated for cancer patients and are effective in identifying patients at nutritional risk who may benefit from nutritional treatment [17]. Unfortunately, they are still rarely used [18]. About 50% of malnourished patients still do not receive adequate nutritional support, which may be, at least in part, due to the fact that the attitude toward the issue of nutritional support for cancer patients varies greatly among oncologists. Insufficient awareness and lack of organized teamwork between oncologists and clinical nutritionists are among the key critical factors that need to be addressed to improve cancer care. Moreover, difficulties in developing a consistent and harmonized nomenclature and the lack of high-quality, evidence-based randomized clinical trials (RCTs) on the effectiveness of nutritional interventions are among the obstacles to the proper detection and treatment of malnutrition and cancer cachexia. In the article by Caccialanza et al. on Italian centers, the daily work of medical professionals in identifying malnutrition was assessed, showing that only 16% of oncology departments routinely used validated tools for assessing nutritional status. The percentage of nutritional support managed by nutritionists was also disappointing—it was provided only in 31% of oncology departments.

However, taking into account that the frequency of eating disorders in patients with pancreatic cancer is very common [19], it seems advisable to refer each patient with pancreatic cancer to a specialist in clinical nutrition (clinical nutritionist) in order to implement prophylactic nutrition monitoring and/or nutritional counseling.

In accordance with the guidelines of the European Society for Clinical Nutrition and Metabolism (ESPEN) on the nutrition of cancer patients, it is recommended to periodically assess the quality and quantity of nutrient intake and the changes in body weight (body mass index—BMI) during cancer diagnosis, as well as to perform assessment and reassessment of nutrition during the treatment [9].

In 2018, the Global Leadership Initiative on Malnutrition (GLIM) criteria for diagnosis of malnutrition were published [20]. Several studies reported the association between GLIM-defined malnutrition and survival [21,22,23]. The GLIM criteria have been validated for pancreatic cancer as well [24].

The Global Leadership Initiative on Malnutrition (GLIM) [5] recommended a two-step strategy for assessing malnutrition. The first step is to identify people “at risk of malnutrition” by using any of the various validated screening tools, and the second step is to determine the degree and severity of malnutrition [5]. A combination of at least one phenotypic criterion (unintentional weight loss, BMI, and muscle loss) and an etiological criterion (decreased food intake, inflammation) is recommended for the diagnosis of malnutrition. Its severity is then classified as moderate (stage 1) or severe (stage 2) [5].

Various screening tools for examining nutritional status have been developed and validated to identify patients at risk of malnutrition, e.g., SGA, MUST, and NRS 2002 [9]. These tools, in conjunction with other parameters, including BMI and other serum markers, e.g., albumin and prealbumin, may help manage strategies for improving the nutritional status of patients with pancreatic cancer [5]. Among the various screening tools, the Malnutrition Universal Screening Tool (MUST) has been validated with high sensitivity and specificity for predicting postoperative morbidity [25]. A higher MUST score is associated with increased morbidity and mortality in PC patients [4]. MUST is a five-step tool that is used in nutritional research and is designed to diagnose malnutrition among adults or those at risk of malnutrition [26]. 

This tool is recommended for use in patients during hospitalization. MUST assesses the patient’s body mass index (BMI), evaluates the percentage of body weight lost in the last 3–6 months, and assesses whether the patient is unable to eat for more than 5 days, which means that the patient receives an additional two points. Then, all of the points are summed up; a score of zero points indicates a low risk of malnutrition, a score of one point indicates a medium risk of malnutrition, and a score of two points or more indicates a high risk of malnutrition. A Spanish group of experts in surgery, radiotherapy, nutritional therapy, and oncology recommended this tool as a screening tool for assessing the nutritional status of patients with PC. Table 2 contains information on further decisions made in patients depending on the obtained MUST score.

According to the recommendations of ESPEN and ASPEN (American Society of Nutritional Treatment and Metabolism), each patient with pancreatic cancer admitted to the hospital should have a screening assessment of their nutritional status, and in the case of malnutrition, they should have a detailed assessment. The aforementioned Nutrition Risk Score (NRS 2002) or Subjective Global Assessment (SGA) scales are recommended [9]. The NRS 2002 and SGA scales are discussed in Table 3.

The International Study Group on Pancreatic Surgery (ISGPS) recommends the measurement of nutritional status as part of the routine preoperative assessment in pancreatic cancer; here, in addition to weight loss and body mass index, it is necessary to measure sarcopenia and sarcopenic obesity because they are strong predictors of unsatisfactory short- and long-term outcomes. Body composition and, in particular, the measurement of muscle mass and visceral adipose tissue cannot be accurately determined with the classical subjective assessment of malnutrition because the proportions of these body parts may be abnormal in malnourished patients, as well as in normal-weight patients and even in obese patients [27,28]. It should be remembered that patients diagnosed with obesity by using BMI without a body composition assessment may still have sarcopenia. It has been proven that a loss of muscle mass before surgery results in poorer outcomes after pancreatic resection. It is very important when assessing a patient’s nutritional status before surgery, in addition to assessing body weight and BMI, to assess body composition and whether the patient has sarcopenic obesity or sarcopenia because they are predictors of poorer outcomes in patients after surgery [29]. It has been proven that the loss of muscle mass significantly affects the hospitalization time, making it longer and increasing the risk of postoperative pancreatic fistula. It has been shown that “sarcopenic obesity”, i.e., reduced muscle mass and excess visceral fat in the abdominal cavity, is a significant factor that increases the risk of mortality associated with surgery [27,30,31,32,33]. 

Considering the long-term outcomes, patients with sarcopenia live for a shorter time than patients without sarcopenia [34,35,36], and the development of sarcopenia has been associated with earlier disease recurrence [33] and with poorer tolerance to adjuvant chemotherapy [37].

Well-defined limits for lean muscle mass and visceral fat have been widely validated in cancer patients, and quantitative abdominal computed tomography (CT) has been recognized as the gold standard for assessing muscle mass and fat mass in cancer patients. It allows for the quantification of different body compartments without performing other body composition tests [38].

A recent meta-analysis [39] assessed the impact of the CONUT (CONTROLLING NUTritional status) screening tool on the prognosis of patients with pancreatic cancer. The CONUT score is calculated with the following parameters: serum albumin, total lymphocyte count, and total cholesterol. Nutritional statuses with scores of 0–1, 2–4, 5–8, and 9–12 are normal, mild, moderate, and severe, respectively. The higher the CONUT score, the worse the nutritional status. The CONUT score can be used as an effective prognostic factor in clinical practice. It is not fully understood how biological mechanisms influence the prognostic value of the CONUT score in relation to poor OS in pancreatic cancer patients, but it can be explained as follows [39]. Serum albumin is a recognized indicator of nutritional status and is related to systemic inflammation [40]. It has been proven that poor survival of cancer patients is related to hypoalbuminemia before treatment [41]. Secondly, low lymphocyte counts in cancer patients may lead to poor survival by weakening the immune response [42]. Thirdly, a level of cholesterol below the norm may affect the anticancer activity of immunocompetent cells [43]. Therefore, the combination of low cholesterol, low serum albumin, and low lymphocyte counts may be associated with poor survival of pancreatic cancer patients and low CONUT scores. Review of the literature on tools for assessing nutritional status in patients with pancreatic cancer is presented in Table 4.

## 5. Nutritional Treatment

### 5.1. Nutritional Counseling

Nutritional counseling should be the first step of the nutritional intervention in patients before treatment for pancreatic cancer is commenced, during the treatment, and after the treatment. An analysis of a patient’s diet by a qualified nutritionist with experience in working with a cancer patient and the detection of deficiencies in the intake of nutrients, vitamins, and minerals, as well as assistance in changing diets to make up for these nutritional deficiencies, is the first step in reducing the risk of developing malnutrition or other eating disorders and their further aggravation [51]. It has been proven that early dietary counseling among patients with pancreatic ductal adenoma (PDAC) improves not only the nutritional status, but also survival [52]. The Enhanced Recovery After Surgery (ERAS) guidelines also recommend an oral diet as a routine nutritional strategy for patients after pancreatoduodenectomy (PD) [53].

The ESPEN guidelines recommend an energy intake of 25–30 kcal/kg/day and a protein intake above 1.0 g/kg/day or, if possible, up to 1.5 g/kg/day [54]. Protease supplements should be considered in the case of high-protein diets. In a prospective cohort study on protein intake after initiation of chemotherapy in patients with nonresectable pancreatic cancer, a protein intake of <1.1, which is at the lower end of the recommended intake, was identified as a poor independent prognostic factor in patients with nonresectable pancreatic cancer [54]. Studies have confirmed that insufficient protein intake leads to poorer clinical outcomes and that effective nutritional intervention to meet protein needs should be undertaken as early as possible.

In general, cancer patients’ diet should be as normal as possible, and fat restriction and high-fiber diets should be avoided. Small, frequent, high-calorie meals are generally recommended because they are easier to digest than large meals.

Muscle depletion is considered a poor prognostic factor in patients with advanced pancreatic cancer [54]. Since adequate protein intake is essential for the maintenance of normal muscle mass, it is suspected that the effect of protein intake on survival may be related to the preservation of muscle mass. Therefore, further studies are needed to explain the mechanisms underlying the beneficial effects of the intake of >1.1 g/kg/day of protein on survival in patients with pancreatic cancer.

### 5.2. Oral Nutritional Supplements

Since loss of appetite and consequent reduced calorie and protein intake are common symptoms in PC patients, the use of oral nutritional supplements (ONSs) may be indicated as an effective strategy for nutritional support and as a second step after nutritional counseling in patients with pancreatic cancer with difficulties in achieving 100% of macronutrients and micronutrient needs from their diet alone [26]. ONSs are used by about 20–55% of patients with pancreatic cancer [26]. They should be regularly reviewed to assess their effectiveness and control possible side effects, and the dosage should be adjusted, as in the case of any medication [26]. Specialists responsible for the treatment of patients with pancreatic cancer must undergo nutritional training in order to prescribe and properly monitor the use of ONSs in patients with PC.

Moreover, the use of ONSs seems to have a direct impact on malnutrition. Two studies have shown significant reductions in Patient-Generated Subjective Global Assessment (PG-SGA) scores after eight weeks of intervention with high-energy, high-protein ONSs.

Worldwide, studies focusing on nutritional interventions in gastrointestinal (GI) cancers have shown mixed results. A systematic review of patients with gastrointestinal (gastric, esophageal, pancreatic) cancer undergoing surgery found little evidence of the effectiveness of ONSs in terms of weight gain and increased energy intake in both the preoperative and postoperative periods [55]. Another recent meta-analysis on the role of oral supplementation with a product enriched with amino acids containing glutamine, vitamins, and minerals during chemotherapy and/or radiotherapy in 445 patients with GI and head and neck cancers showed that this type of nutritional intervention may be beneficial in preventing chemotherapy-related toxicity [56]. 

A prospective randomized study conducted among patients with pancreatic cancer who received neoadjuvant chemoradiation therapy (NACRT) showed that the consumption of dietary supplements enriched with eicosapentaenoic acid (EPA) may have a beneficial effect on improving the nutritional status of patients. Nutritional interventions implemented among patients with other malignant tumors also had a positive effect on the improvement of body composition parameters and body weight [56,57]. However, the data are still ambiguous as to the appropriate type of nutritional intervention, and there is a need for large-scale multicenter studies on the impacts of nutritional treatment on outcomes, quality of life, the occurrence of treatment side effects, and survival of patients with pancreatic cancer.

### 5.3. Pancreatic Enzyme Replacement Therapy (PERT)

One of the main factors contributing to the development of malnutrition or other eating disorders in cancer patients is the development of PEI. The use of pancreatic enzyme replacement therapy by patients in order to reduce the occurrence of side effects, e.g., digestive problems, steatorrhea, and abdominal pain, is, apart from individualized dietary counseling, the basis of nutritional treatment in this group of patients, and it can also significantly improve their nutritional status.

Pancreatic exocrine insufficiency (PEI) may be caused by local lesions that are caused by a tumor or accompanying inflammation, or it may be a consequence of surgery [58]. The incidence of PEI after pancreatic cancer surgery is reported in 50–100% of patients after pancreatic duodenectomy (PD), which is also called the Whipple procedure [59], and in 0–42% of patients after distal pancreatectomy (DP) [59].

The primary management strategy for PEI is the use of pancreatic enzyme replacement therapy (PERT). Regardless of the extent of pancreatic resection or surgical technique, patients should be evaluated for endocrine and exocrine pancreatic insufficiency. PERT should probably be started routinely after PD and continued for at least 6 months after surgery. PERT should also be initiated in post-DP patients with symptoms of PEI. Patients with untreated PEI may develop complications such as weight loss, poor wound healing, deficiencies of fat-soluble vitamins, and electrolyte imbalances, which should be closely monitored [29].

The dosage of pancreatic enzymes should be started with low doses and then increased every few days depending on the fat content in diet, stool characteristics, and each patient’s symptoms [58].

Dosage suggestions vary, but they typically start at 20,000–75,000 lipase units per meal and 5000–50,000 lipase units per snack [58]. PERT doses should not exceed 10,000 lipase units per kilogram of body weight per day or 2500 lipase units per kilogram per meal, up to four times a day [58]. Such large differences in the dosage range are related to the fact that patients who often experience side effects related to digestive problems limit their food intake and limit the amount of fat in their diet. This may lead to a faster development of malnutrition in this group of patients because some cancer patients have a greater need for energy and protein from the diet, while limiting the consumption of meals may lead to the loss of muscle mass and the development of eating disorders.

When deciding on the initial dosage of pancreatic enzymes, it is important that the patient’s diet be taken into account by a specialist who recommends the use of pancreatic enzymes. With adequate supplementation of pancreatic enzymes, there is no need to limit the dietary fat intake. Dietary fat intake that is lower than required may be beneficial in patients with severe steatorrhea. Sarner recommends that the consumption of fat should be below 75 g per day [60].

However, it should be remembered that a “healthy diet” based on the recommended daily intake of macronutrients, including fat, probably provides <75 g of fat per day (in an adult consuming 2000 kcal/day, 30% of the total energy intake should come from fat, so they would consume only 67 g of fat per day). For patients that have trouble consuming the adequate/correct amounts of calories due to a limited ability to digest and absorb fat, medium-chain fatty acids (MCTs) constitute the recommended source of dietary fat because MCTs do not require enzymatic action for digestion or absorption. Appropriate dosing of PERT is critical in the proper digestion and absorption of fats in patients with PEI, but is also plays a role in improving or maintaining a normal quality of life, as it is possibly related to improved bowel function and reduced diarrhea and steatorrhea.

In a double-blind trial of patients with nonresectable PC, patients that were randomized for the use of PERT had better absorption of dietary fat, and their weight loss was inhibited compared to that of the placebo group [61]. In another retrospective non-randomized study of patients with nonresectable PC receiving PERT plus standard palliative care or standard palliative care alone, the median survival of patients receiving PERT was longer than that of patients receiving standard palliative care (301 versus 89 days) [51]. Another retrospective and observational study conducted in the UK with a large sample of patients showed that the median survival time was 262% longer in patients undergoing PERT compared to that in patients not undergoing PERT [62].

However, PERT is not always used in practice, and enzyme doses are often lower than needed [63]. In a retrospective study of 4554 PC patients, only 21.7% were prescribed PERT [62]. Due to the complex etiology of malnutrition in PC patients, special attention should be paid to every aspect that can improve the nutritional status, and PERT must constitute part of the nutritional intervention.

### 5.4. Enteral (EN) and Parenteral (PN) Nutrition

Patients with pancreatic cancer who, despite dietary counseling, are unable to meet their total energy and other nutritional requirements via oral intake or ONSs and who have normal bowel function require total or complementary enteral nutrition. Enteral nutrition is recommended as a first-line method in this group of patients. When enteral nutrition is used, the principle of supplying food to the most efficient section of the digestive tract applies. Intragastric access is considered first, followed by enteral access. Intragastric access is achieved via the insertion of a nasogastric tube through the nose if the duration of the planned intervention does not exceed 30 days or through percutaneous endoscopic gastrostomy (PEG), which is the gold standard. Although patients with PC prefer gastrostomy to nasogastric tubes, tube feeding is associated with a lower complication rate [63]. However, in patients with pancreatic cancer, intragastric feeding is often ineffective due to gastric emptying disorders.

Therefore, in prehabilitation parallel to postoperative neoadjuvant therapy, a nasoenteric tube is a better solution. However, after surgical treatment of patients, a nutritional jejunostomy introduced during surgery in order to accelerate postoperative rehabilitation and to carry out adjuvant chemotherapy for a period of time is the access of choice. Enteral nutrition is usually ineffective in palliative patients due to multilevel transit disorders. 

Patients with pancreatic cancer undergoing prehabilitation for surgery with access to the gastrointestinal tract are qualified for enteral nutrition and should have a nasogastric tube or a nutritional jejunostomy. On the other hand, patients with pancreatic cancer in the case of which access to the gastrointestinal tract cannot be obtained are qualified for parenteral nutrition. Patients with pancreatic cancer treated with chemotherapy and patients with gastrointestinal insufficiency receive parenteral nutrition, and patients whose gastrointestinal tract functions properly receive enteral nutrition.

Total parenteral nutrition (TPN) may be used when EN is insufficient or contraindicated. TPN may improve body weight in some patients before surgery, but it is not routinely recommended in outpatient palliative care due to the rapid progression of cancer and the many health problems that usually accompany it, as these require inpatient treatment. Careful evaluation should be carried out to identify patients who might benefit from this nutritional method. Life expectancy and ethical aspects should be taken into account. Therefore, the use of TPN must be an individualized and multidisciplinary decision of physicians and nutritionists [51].

Malignant bowel obstruction frequently occurs in the end stage of pancreatic cancer. Parenteral nutrition should be considered in patients at this stage [64].

Emanuel et al. [15] carried out a systematic review and investigated the effects of various nutritional interventions in the treatment of cachexia, malnutrition, and weight loss in PC patients. PN was associated with more frequent complications. EN showed a positive effect on length of hospitalization, complications, cytokines, and weight loss. Nutritional supplements enriched with omega-3 fatty acids affected the maintenance of and possible increase in body weight and lean body mass. After considering each intervention, the systematic review concluded that an individualized diet should be used depending on the patient’s condition or, if possible, consisting of EN or nutritional supplements as immunonutrition enriched with omega-3 fatty acids and specific amino acids.

### 5.5. Medical Nutrition Depending on the Stage of Pancreatic Cancer

According to the National Comprehensive Cancer Network (NCCN) guidelines [65], PC is classified as resectable/borderline resectable, locally advanced nonresectable, and metastatic.

The advancement of pancreatic cancer is of fundamental importance in the further treatment process, as well as for the recommendations for the patient’s diet and nutrition. Before the commencement of treatment and between sessions, it is necessary to perform a nutritional status assessment for patients who will undergo neo-adjuvant therapy, radiotherapy and chemotherapy, radiotherapy alone, or chemotherapy alone. A very important element of treatment of a patient with pancreatic cancer is addressing the side effects related to the cancer itself or to the method of treatment. Symptoms such as vomiting, diarrhea, pain, anorexia, PEI, and others may worsen the patient’s nutritional status. 

In patients who are qualified for surgery, it is necessary to assess the nutritional status and diet before surgery, and preoperative patients at risk of malnutrition should be advised to use dietary supplements (ONSs) before surgery for at least 5–7 days. In severely malnourished patients, delaying surgery by 7 to 14 days should be considered, and in patients who are unable to successfully receive oral or enteral nutrition, total or supplemental parenteral nutrition may be considered to improve their nutritional status. The patients should be included in the ERAS (Enhanced Recovery after Surgery) protocol. After surgery, it is recommended to try to gradually introduce oral nutrition as soon as possible in the case of postoperative complications that make oral/enteral nutrition difficult, and if it is not possible to cover the energy demand, an appropriate strategy is to use early TPN nutrition [26].

Approximately 70% of PC patients are diagnosed with metastases [66,67]. Systemic chemotherapy is considered the standard of care in this group of patients and has shown a significant improvement in survival, but it is associated with many side effects that lead to dose reduction and delay or discontinuation of treatment [68]. It is essential to assess the nutritional status of these patients to limit its impact on the severity of side effects and treatment discontinuation [67,68].

Finally, in the case of palliative patients, nutritional therapy must be individualized for each patient so that the patient achieves comfort and quality of life. Many strategies can be used for this purpose, but currently, there is no clear consensus, so they may differ and encompass the use of oral nutritional supplements, enteral nutrition, or, in selected cases, TPN.

### 5.6. Preoperative Nutritional Support

It is advisable to carry out a thorough assessment of the patient’s nutritional status in the preoperative period. Currently, there are no studies or guidelines supporting universal preoperative nutritional therapy. Although the benefits of nutritional support have been demonstrated in randomized controlled trials (RCTs), benefits have only been documented in patients with severe malnutrition or at high risk of developing malnutrition who were administered parenteral or enteral nutrition for at least 7 days before surgery. Based on these results, preoperative nutritional support should be seriously considered if at least one of the following criteria is met:

(1) Weight loss (WL) > 15% in 6 months;
(2) BMI < 18.5 kg/m^2^;
(3) Subjective global assessment (SGA), grade C or assessment of risk related to the nutritional status;
(4) Serum albumin < 30 g/L.

Enteral nutrition is always recommended after surgery to maintain intestinal transit and barriers and to possibly reduce infection. Initiation of oral nutrition as soon as possible after surgery was associated with a shorter hospitalization time in one observational study, with no differences in morbidity. Oral nutrition did not worsen the course, even in patients with pancreatic fistula [69].

On the other hand, parenteral nutrition may be considered when oral nutrition is not possible, e.g., in patients with delayed gastric emptying, paralytic ileus, or anastomosis complications. When enteral nutrition is impossible, it is necessary to start parenteral nutrition; it is important to start it as soon as possible after surgery—the recommendation is within 3 days of surgery. PERT treatment in patients after pancreatectomy is also routinely recommended. It should be remembered that after surgery, fecal elastase will be low and may not be a good diagnostic marker. Therefore, it is important to detect the symptoms of PEI and choose the correct PERT dosage. Vitamin B12 deficiency may also occur in patients who underwent PD. The development of diabetes is related to the size of the resected pancreas and does not always occur [4].

## 6. Conclusions

In this literature review, the tools that have been applied to assess nutritional status were evaluated, and the knowledge on the current nutritional treatment of patients with pancreatic cancer was reviewed. Assessing the nutritional status at diagnosis and during treatment is essential in the case of this type of cancer. There is a small number of studies on nutritional status assessment tools in pancreatic cancer, most of which are retrospective studies. There is a need to conduct multicenter prospective studies on the evaluation of tools for assessing nutritional status in patients with pancreatic cancer. Each patient diagnosed with pancreatic cancer requires an assessment of his/her nutritional status before starting treatment, but also during treatment, and nutritional counseling should be provided by a qualified nutritionist in order to develop an individualized diet depending on the patient’s condition or to include dietary supplements (ONSs) as immunonutrition enriched with omega-3 fatty acids. If oral nutrition is impossible or incomplete, enteral nutrition should be considered. However, after patients’ surgical treatment, the access of choice is a nutritional jejunostomy that is introduced during surgery to accelerate postoperative rehabilitation. If a patient has contraindications for enteral nutrition, parenteral nutrition should be considered. PERT should be initiated earlier and at a higher dose, since prolonged survival has been observed when applying PERT. Ensuring adequate nutrient intake improves quality of life and chemotherapy tolerance in these patients. However, despite solid evidence linking nutritional status and clinical outcomes, attitudes towards nutritional care continue to vary among oncologists, surgeons, and nutritionists, and a significant percentage of patients do not undergo nutritional assessment or nutritional treatment.

Nevertheless, further well-designed studies are needed to assess the real impact of nutritional support during the treatment of patients with pancreatic cancer and to determine the most effective strategy for reducing the percentage of patients diagnosed with malnutrition, which significantly affects the prognosis and effectiveness of nutritional treatment of patients with pancreatic cancer.

## Figures and Tables

**Table 1:** Degrees of malnutrition severity [13].

(1) Precachexia (includes early clinical and metabolic symptoms preceding significant, involuntary weight and muscle loss):	- weight loss < 5% + anorexia and metabolic changes
(2) Cachexia	- weight loss > 5% - BMI < 20 and weight loss > 2% - sarcopenia and weight loss > 2%
(3) Advanced (irreversible) cachexia	- variable degree of cachexia - ineffective cancer treatment - increased catabolism - low overall performance - expected survival < 3 months

**Table 2:** Decision-making strategy according to the MUST score.

MUST = 0	Control and observation by the department of oncology or surgery.	MUST = 0 (no risk of malnutrition). Nutritional assessment every 2 months to check whenever there are any clinical changes that may adversely affect the nutritional status of patients.
MUST = 1	Nutritional control and treatment by the department responsible for the treatment of patients, starting with nutritional counseling and oral nutritional supplements (ONSs). The patient must be periodically reassessed.	MUST = 1 (moderate risk of malnutrition): assess nutritional status within 2–3 weeks.
MUST ≥ 2	The patient must be referred to the nutrition department.	MUST ≥ (high risk of malnutrition): assessment within 5–7 days.

**Table 3:** Description of the NRS 2002 (nutrition risk score) and the SGA (subjective global assessment).

**Nutrition Risk Score—NRS 2002**
The test consists of the evaluation of four elements: Is the patient’s BMI below 20.5? Has the patient lost weight in the last 3 months? Has the patient eaten less in the last week? Is the patient in a serious condition (e.g., in the intensive care unit)?
If the answer to any question is positive, proceed to the second part of the study. If all answers are negative, the examination should be performed in a week.
**NRS 2002 Part 2**
One appropriate degree of nutritional status disorder and disease severity should be selected. If the sum of the points is: ≥3—the treating physician should be notified, and a nutritional intervention should be implemented. ≤3—malnutrition is unlikely; repeat assessment in 7 days.
**Subjective Global Assessment—SGA**
The test consists of evaluating the following elements: - Age, height, body weight - Changes in body weight - Changes in food intake - Gastrointestinal symptoms - Physical fitness - Disease vs. the need for nutrients - Physical examination
If the SGA result is A, the assessment should be carried out in a week; if the SGA result is B or C, then detailed assessment of the nutritional status should be performed immediately.

**Table 4:** Review of the literature on tools for assessing nutritional status in patients with pancreatic cancer.

Author	Year	Methodology	Results	Conclusions
**Kato et al. [44]**	**2018**	Retrospective studies	The high-risk CONUT group had significantly lower overall survival than the low-risk CONUT group. The CONUT score had an independent relationship with overall survival. The CONUT score showed no association with postoperative pancreatic fistula or postoperative hospitalization.	The CONUT score was independently associated with survival in patients with PDAC after pancreatectomy and was not associated with recurrence or postoperative complications.
**Menozzi et al. [45]**	**2023**	Retrospective studies	Weight loss affected postoperative morbidity/mortality, and decreased muscle mass was an independent predictor of postoperative peptic hemorrhage. There was no relationship between the parameters of nutritional status before surgery and the length of hospitalization, 30-day re-intervention, pancreatic fistula, biliary fistula, and delayed gastric emptying.	Impaired nutritional status prior to pancreatic surgery affects many postoperative outcomes. Measurement of nutritional status supported by CT analysis of body composition parameters, especially muscle mass, should be the gold standard of preoperative assessment in order to obtain early and appropriate nutritional support.
**Vashi et al. [46]**	**2015**	Retrospective studies	SGA was an independent predictor of survival. Patients with lower SGA had a risk of death that was 1.5 times greater than that of patients with higher SGA.	The improvement in SGA during PC treatment was correlated with a significantly reduced risk of mortality regardless of gender, history of prior treatment, and evidence of antitumor biological activity. Maintaining or improving nutritional status during pancreatic cancer treatment was associated with better outcomes.
**Zhou et al. [47]**	**2022**	Prospective studies	Phase angle (PhA) value: The values in the nutritional risk group and the malnourished group were significantly lower than those in the properly nourished group. PhA was positively correlated with nutritional status. The PhA value of the group with postoperative complications was significantly lower than that of the group without complications.	PhA was associated with nutritional status and can be considered a tool for assessing nutritional status in patients with pancreatic head cancer and predicting postoperative complications in patients who have undergone PD.
**Mao et al. [48]**	**2020**	Retrospective studies	Both a low PNI (≤45) and a high CONUT (≥3) were independent risk factors for poor overall survival. CONUT may have greater sensitivity and specificity in predicting complications and survival.	Preoperative low PNI (≤45) and high CONUT scores (≥3) may be reliable predictors of prognosis and surgical complications in patients with PDAC. Compared to PNI, CONUT can be more effective.
**Phillips et al. [49]**	**2022**	Systematic review	Studies were mainly limited by retrospective designs. A meta-analysis could not be performed due to heterogeneity in study design and reporting methods. Patients with PC had a deterioration of their nutritional status, and 44–63% of patients undergoing neoadjuvant chemotherapy had low muscle mass before starting treatment.	There is a shortage of data on nutritional intervention in pancreatic cancer. Future work should include the use of validated functional and clinical assessment tools to further explore the impact of nutritional intervention and the relationship between nutritional status and outcomes.
**Jabłońska et al. [50]**	**2021**	Retrospective studies	Higher nutritional risk according to NRS 2002 was significantly associated with older age, greater weight loss, lower BMI, lower total lymphocyte count, longer hospitalization, neoadjuvant chemotherapy, and preoperative biliary drainage. Low PNI was significantly associated with greater body weight loss, lower total serum protein and albumin concentrations and lymphocyte counts, higher neutrophil/lymphocyte counts (NLR), and length of hospitalization.	Eating disorders were correlated with a systemic inflammatory response in PC patients. Obesity (BMI ≥ 30 kg/m^2^) and malnutrition (NRS 2002 ≥ 3) predicted postoperative complications that were associated with a longer hospitalization.

## Data Availability

Data available from the authors upon request.
